# Low dose native type II collagen prevents pain in a rat osteoarthritis model

**DOI:** 10.1186/1471-2474-14-228

**Published:** 2013-08-01

**Authors:** Lorenzo Di Cesare Mannelli, Laura Micheli, Matteo Zanardelli, Carla Ghelardini

**Affiliations:** 1 Department of Neurosciences, Psychology, Drug Research and Child Health - Neurofarba - Pharmacology and Toxicology Section, University of Florence, Viale Pieraccini 6, 50139, Florence, Italy

**Keywords:** Monoiodoacetate, Oral tolerance, CTX-II, Articular pain

## Abstract

**Background:**

Osteoarthritis is the most widespread joint-affecting disease. Patients with osteoarthritis experience pain and impaired mobility resulting in marked reduction of quality of life. A progressive cartilage loss is responsible of an evolving disease difficult to treat. The characteristic of chronicity determines the need of new active disease modifying drugs. Aim of the present research is to evaluate the role of low doses of native type II collagen in the rat model of osteoarthritis induced by sodium monoiodoacetate (MIA).

**Methods:**

1, 3 and 10 mg kg^-1^ porcine native type II collagen were daily per os administered for 13 days starting from the day of MIA intra-articular injection.

**Results:**

On day 14, collagen-treated rats showed a significant prevention of pain threshold alterations induced by MIA. Evaluation were performed on paws using mechanical noxious (Paw pressure test) or non-noxious (Electronic Von Frey test) stimuli, and a decrease of articular pain was directly measured on the damaged joint (PAM test). The efficacy of collagen in reducing pain was as higher as the dose was lowered. Moreover, a reduced postural unbalance, measured as hind limb weight bearing alterations (Incapacitance test), and a general improvement of motor activity (Animex test) were observed. Finally, the decrease of plasma and urine levels of CTX-II (Cross Linked C-Telopeptide of Type II Collagen), a biomarker of cartilage degradation, suggests a collagen-dependent decrease of structural joint damage.

**Conclusions:**

These results describe the preclinical efficacy of low dosages of native type II collagen as pain reliever by a mechanism that involves a protective effect on cartilage.

## Background

Osteoarthritis pathophysiology involves the whole joint in a disease process that includes focal and progressive loss of hyaline articular cartilage. Concomitant changes in the bone underneath the cartilage involve formation of osteophytes and bony sclerosis, as well as alterations in the synovium and joint capsule [1]. Cartilage loss or degeneration may be a result of natural aging, obesity, repeated trauma or hormone disorders. The mechanical stress on the damaged joint irritates and inflames the cartilage causing joint pain and swelling [2,3].

An integrative treatment of osteoarthritis, or rheumatoid arthritis, must consider a supplementation with collagen since it is the most prevalent component of the solid phase of articular cartilage [4]. The three major groups of collagen derivatives clinically used for arthritis treatment are based on the various degrees of hydrolysis of collagen: gelatin, collagen hydrolysate and native undenatured collagen [5]. Analogous working mechanisms has been described for gelatin and collagen hydrolysate: after oral administration peptides can be used as building blocks for the cartilage [6-8]. Moreover, it is hypothesized that collagen hydrolysate also influences bone metabolism [9,10] or the vascular system involved in the atheromatous disease of the subchondral bone [11,12]. For these purposes collagen hydrolysate is dosed in grams *per* day (usually 10 g) [13-15]. On the contrary, undenatured collagen has been reported as beneficial for articular pain when per os administered in the order of milligrams [16,17]. Undenatured collagen was preclinically and clinically studied mainly in rheumatoid arthritis [16,17]; the autoimmune component of this pathology suggests a mechanism called oral tolerance, the usual response of the gut-associated lymphoid tissue (GALT) to harmless gut antigens inducing local and systemic immunological tolerance [18-20]. The knowledge about the relevance of low doses of undenatured collagen in osteoarthritis are more limited [21] and the absence of an immune component in the pathology of osteoarthritis make difficult to assume the oral tolerance as possible mechanism of collagen action.

In order to verify the efficacy of low doses of porcine native type II collagen as pain reliever and cartilage protector, we determined its pharmacological profile in a rat unilateral osteoarthritis induced by sodium monoiodoacetate (MIA).

## Methods

### Animals

For all the experiments described below, male Sprague-Dawley rats (Harlan, Varese, Italy) weighing approximately 200-250 g at the beginning of the experimental procedure were used. Animals were housed in CeSAL (Centro Stabulazione Animali da Laboratorio, University of Florence) and used at least one week after their arrival. Four rats were housed per cage (size 26 × 41 cm); animals were fed with standard laboratory diet and tap water *ad libitum*, and kept at 23 ± 1°C with a 12 h light/dark cycle, light at 7 a.m. All animal manipulations were carried out according to the European Community guidelines for animal care (DL 116/92, application of the European Communities Council Directive of 24 November 1986 (86/609/EEC). The ethical policy of the University of Florence complies with the Guide for the Care and Use of Laboratory Animals of the US National Institutes of Health (NIH Publication No. 85-23, revised 1996; University of Florence assurance number: A5278-01). Formal approval to conduct the experiments described was obtained from the Animal Subjects Review Board of the University of Florence. Animals were anaesthetised before cervical dislocation. All efforts were made to minimize animal suffering and to reduce the number of animals used.

### Monoiodoacetate-induced osteoarthritis

Unilateral osteoarthritis was also induced by injection of monoiodoacetate (MIA, Sigma-Aldrich) into the knee joint according to a described method [22,23]. On day 1, rats were slightly anesthetized by 2% isoflurane, the left leg skin was sterilized with 75% ethyl alcohol and the knee located by palpation; then, a 28-gauge needle was inserted vertically to penetrate the skin and turned distally for insertion into the articular cavity until a distinct loss of resistance was felt. 2 mg MIA in 25 μl saline were delivered into the left articular cavity. Control rats received 25 μL of saline solution (day 1) in the knee joint. Behavioral and biochemical measures were performed at day 14.

### Drug treatments

Porcine native type II collagen (Bioiberica, Spain) was suspended in 1% carboxymethylcellulose sodium salt (CMC) and administered by the *per os* (p.o.) route. 1, 3 or 10 mg kg^-1^ collagen were daily administered starting from the day 1 immediately after MIA injection to the 13^th^ day. Behavioural and biochemical tests were performed 24 hours after the end of treatments. Control rats received p.o. CMC every day. For all tests blind experiments were performed.

### Paw Pressure test

The nociceptive threshold in the rat was determined with an analgesimeter (Ugo Basile, Varese, Italy), according to the method described by [24]. Briefly, a constantly increasing pressure was applied to a small area of the dorsal surface of the paw using a blunt conical probe by a mechanical device. Mechanical pressure was increased until vocalization or a withdrawal reflex occurred while rats were lightly restrained. Vocalization or withdrawal reflex thresholds were expressed in grams. Rats scoring below 40 g or over 80 g during the test before drug administration were rejected (25%). For analgesia measures, mechanical pressure application was stopped at 120 g.

### Von Frey test

The animals were placed in 20 cm x 20 cm plexiglas boxes equipped with a metallic meshy floor, 20 cm above the bench. A habituation of 15 minutes was allowed before the test. An electronic Von Frey hair unit (Ugo Basile, Varese, Italy) was used: the withdrawal threshold was evaluated by applying force ranging from 0 to 50 grams with a 0.2 gram accuracy. Punctuate stimulus was delivered to the mid-plantar area of each anterior paw from below the meshy floor through a plastic tip and the withdrawal threshold was automatically displayed on the screen. Paw sensitivity threshold was defined as the minimum pressure required to elicit a robust and immediate withdrawal reflex of the paw. Voluntary movements associated with locomotion were not taken as a withdrawal response. Stimuli were applied on each anterior paw with an interval of 5 seconds. The measure was repeated 5 times and the final value was obtained by averaging the 5 measures [25].

### PAM test

The Pressure Application Measurement (PAM from Ugo Basile, Italy) was used to measure mechanical pain threshold of the knee jont. A force transducer (2 mm diameter) is mounted on the operator’s thumb and a progressive a quantified force was applied for direct stimulation of the joint. The rate of application of the force is decided by the operator. The force (gram-force; gf) which elicits the animal response (normally, limb withdrawal) was recorded. The value considered for each joint was the mean of 5 consecutive measurements. Data are expressed as the difference between the force tolerated on the knee joint contralateral to the injury and the force tolerated on the ipsilateral one (Δ Force).

### Incapacitance test

Weight bearing changes were measured using an incapacitance apparatus (Linton Instrumentation, UK) detecting changes in postural equilibrium after a hind limb injury [26]. Rats were trained to stand on their hind paws in a box with an inclined plane (65° from horizontal). This box was placed above the incapacitance apparatus. This allowed us to independently measure the weight that the animal applied on each hind limb. The value considered for each animal was the mean of 5 consecutive measurements. In the absence of hind limb injury, rats applied an equal weight on both hind limbs, indicating a postural equilibrium, whereas an unequal distribution of the weight on hind limbs indicated a monolateral decreased pain threshold. Data are expressed as the difference between the weight applied on the limb contralateral to the injury and the weight applied on the ipsilateral one (Δ Weight).

### Spontaneous activity meter (Animex)

Locomotor activity in rats was quantified using an Animex activity meter Type S (LKB, Farad, Sweden) set to maximum sensitivity. Every movement of rats, which were placed on the top of the Animex activity meter, produced a signal due to variation in inductance and capacity of the apparatus resonance circuit. Signals were converted automatically to numbers. On the day of the experiment the cage, containing three rats, were put on the measuring platform. Activity counts were made for 5 min.

### CTX-II and CPII levels

On the day 14 plasma and urine samples were collected and analyzed to measure the Cross Linked C-Telopeptide of Type II Collagen (CTX*-*II) levels. Dosages were performed using ELISA assay (Antibodies online, Germania) by specific antibody. The method was in accordance to the procedure described by [27] for plasma and urine measure, respectively. Plasmatic levels of CPII, the carboxyl propeptide of type II procollagen, were also evaluated by ELISA assay (Ibex, Canada) according to [28,29].

### Statistic analysis

Results were expressed as means ± S.E.M. and the analysis of variance was performed by ANOVA. A Bonferroni’s significant difference procedure was used as post-hoc comparison. *P* values of less than 0.05 or 0.01 were considered significant. Data were analyzed using the “Origin 8.1” software.

## Results

**Figure 1:**
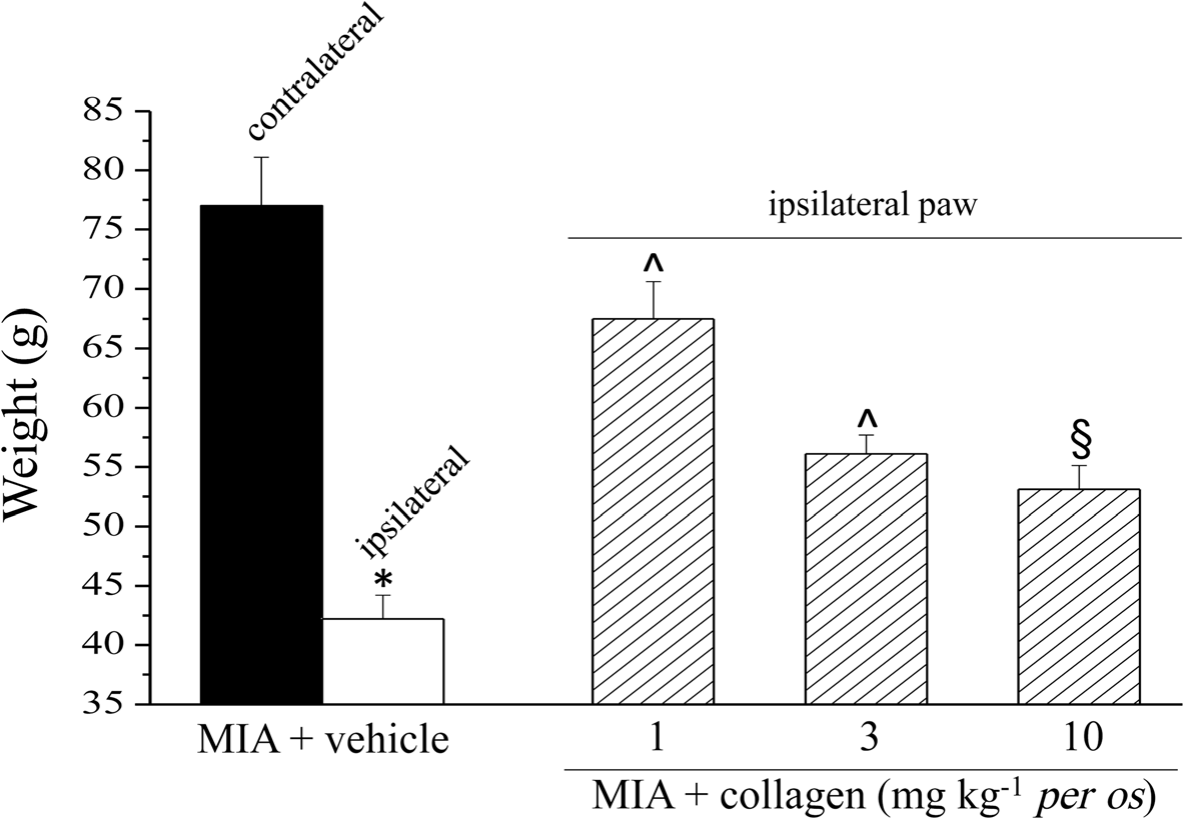
**Pain: noxious stimulus, Paw-pressure test.** Monoarthritis was induced by injection of MIA into the knee joint. On day 1, 2 mg MIA in 25 μl saline were delivered in the left (ipsilateral) articular cavity. Paw pressure test was performed on day 14. Collagen was suspended in 1% CMC and daily administered p.o. starting on the 1^th^ day suddenly after MIA. Control animals were treated with vehicle. Each value represents the mean of 12 rats performed in 2 different experimental set.*P < 0.01 *vs* the contralateral paw; ^§^P < 0.05 and ^P < 0.01 *vs* the MIA + vehicle treated rats.

**Figure 2:**
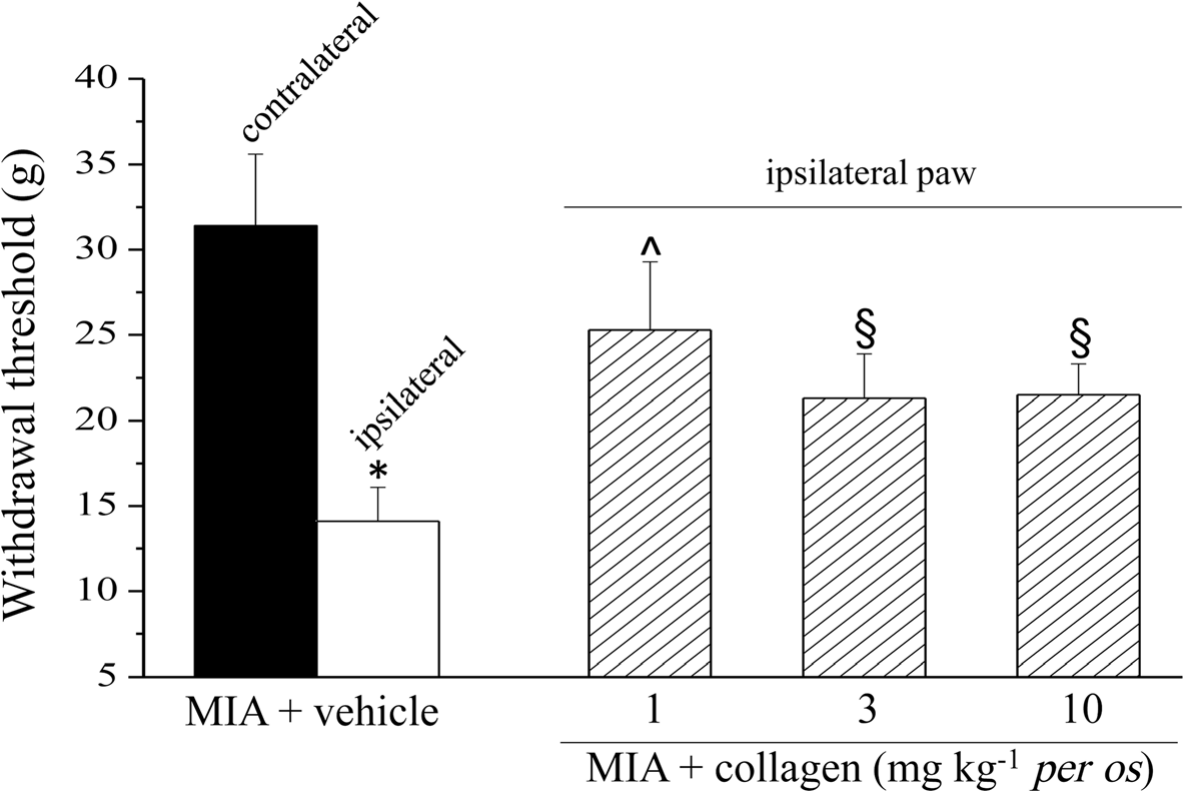
**Pain: non-noxious stimuli.** Von Frey test was used to measure the response evoked by a mechanical stimulus 14 days after MIA injection. 1, 3 or 10 mg kg^-1^ collagen were p.o. administered every day starting on the 1^th^ day suddenly after MIA. Control animals were treated with vehicle. Each value represents the mean of 12 rats performed in 2 different experimental set.*P < 0.01 *vs* contralateral paw; ^§^P < 0.05 and ^P < 0.01 *vs* MIA + vehicle treated rats.

**Figure 3:**
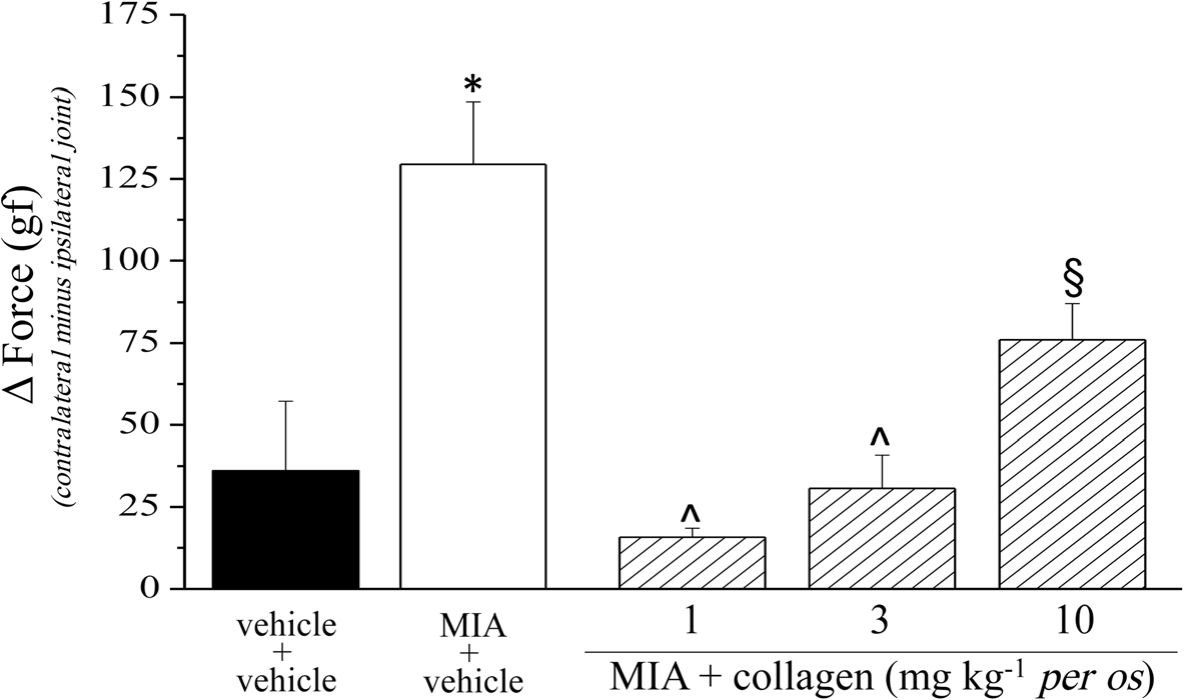
**Articular pain, PAM test.** The effect of collagen (daily administered starting on the 1^th^ day) on MIA-induced articular damage was evaluated on day 14. Data are expressed as the difference between the force tolerated on the knee joint contralateral to the injury and the force tolerated on the ipsilateral one. Control animals were treated with vehicle. Each value represents the mean of 12 rats performed in 2 different experimental set. *P < 0.01 compared to vehicle + vehicle rats; ^§^P < 0.05 and ^P < 0.01 compared to MIA + vehicle treated rats.

The pharmacological activity of low dosages of type II collagen was evaluated in the rat unilateral osteoarthritis model induced by MIA. 14 days after intra-articular MIA injection the mechanical withdrawal threshold to a noxious stimulus was measured by Paw pressure test (Figure 1). The weight tolerated on the ipsilateral paw was significantly reduced (42.2 ± 2.0 g) compared to the contralateral (77.0 ± 4.1 g). 1 mg kg^-1^ collagen administered daily p.o. for 13 days (starting from the day of MIA injection) increased the withdrawal threshold of the ipsilateral paw up to 67.5 ± 3.1 g. The doses of 3 and 10 mg kg^-1^ were significantly active but progressively less effective. Figure 2 shows the response to a non-noxious mechanical stimulus evaluated by the Von Frey test. On day 14 pain threshold of the ipsilateral paw (MIA + vehicle group) was decreased to 14.1 ± 2.0 g as compared to the contralateral (31.4 ± 4.2 g). Animals treated with 1 mg kg^-1^ collagen showed an ipsilateral threshold of 25.3 ± 4.0 g; the groups treated with higher dosage tolerated a stimulus of about 21 g. In both Paw pressure and Von Frey tests the pain sensitivity of the contralateral paw of MIA + vehicle or MIA + collagen groups was not different with respect to the control (vehicle + vehicle, data not shown). Articular pain was evaluated by PAM test, in Figure 3 the difference (Δ Force) between the force tolerated directly on the knee joint contralateral to the injury and the force tolerated on the ipsilateral one was described. Δ Force in control rats (vehicle + vehicle, 36.1 ± 21.1 gf) was dramatically increased on the day 14 in MIA + vehicle group (129.5 ± 10.1 gf). Collagen treatment prevented articular pain in a manner inversely proportional to the dose, reaching 15.8 ± 2.7 gf Δ Force in animals treated with 1 mg kg^-1^.

**Figure 4:**
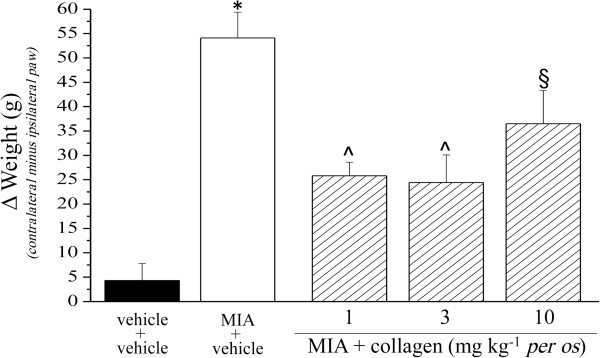
**Hind limb weight bearing alterations, Incapacitance test.** The effect of collagen (daily administered starting on day 1) on MIA-induced articular damage was evaluated on day 14. Data are expressed as the difference between the weight applied on the limb contralateral to the injury and the weight applied on the ipsilateral one. Control animals were treated with vehicle. Each value represents the mean of 12 rats performed in 2 different experimental set. *P < 0.01 *vs* vehicle + vehicle rats; ^§^P < 0.05 and ^P < 0.01 *vs* MIA + vehicle treated rats.

**Figure 5:**
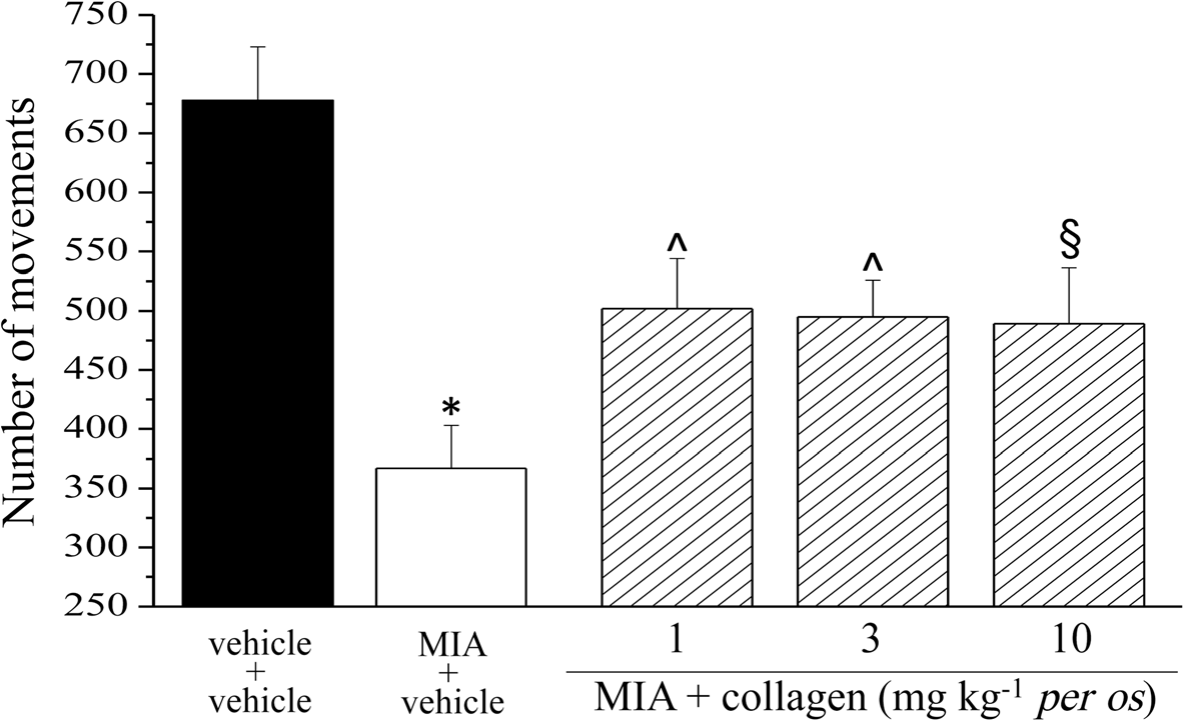
**Motor activity alterations, Animex test.** Motor capability was observed by measuring the number of movements in 5 minutes on a free surface. The test was performed 14 days after MIA injection and effect of daily repeated collagen administration starting from the day 1 was evaluated. Control animals were treated with vehicle. Each value represents the mean of 12 rats performed in 2 different experimental set. *P < 0.01 compared to vehicle + vehicle rats; ^§^P < 0.05 and ^P < 0.01 compared to MIA + vehicle treated rats.

Unilateral pain was also able to induce hind limb weight bearing alterations (Incapacitance test): the difference between the weight burdened on the contralateral and the ipsilateral limb was significantly increased in MIA + vehicle (54.1 ± 5.3 g) with respect to vehicle + vehicle (4.3 ± 3.5). The protective effect of collagen was shown in Figure 4. Moreover, the Animex test showed a collagen-dependent improvement of motor activity increasing the number of movements by about 50% (Figure 5).

**Figure 6:**
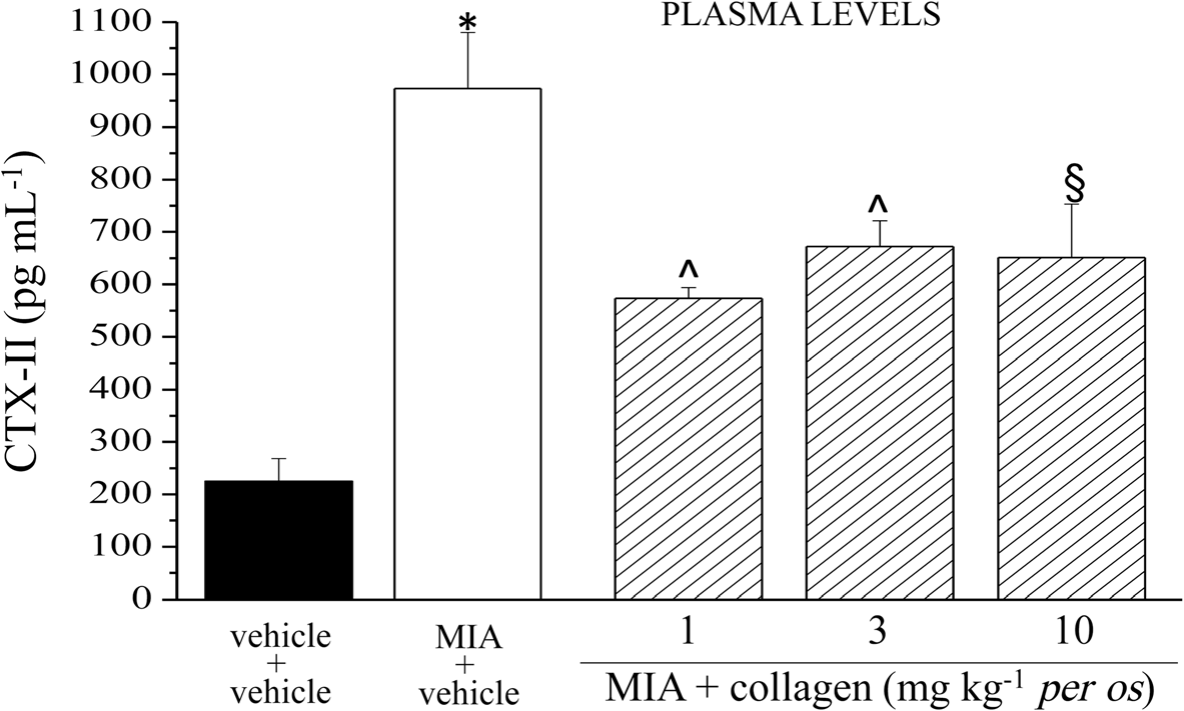
**CTX-II, plasma levels.** On day 1, 2 mg MIA in 25 μl saline were delivered in the left knee articular cavity. Collagen was suspended in 1% CMC and p.o. daily administered starting on day 1. Control animals were treated with vehicle. On day 14 plasma samples were collected and analyzed by ELISA kit. Each value represents the mean of 12 rats performed in 2 different experimental set. *P < 0.01 compared to vehicle + vehicle rats; ^§^P < 0.05 and ^P < 0.01 compared to MIA + vehicle treated rats.

**Figure 7:**
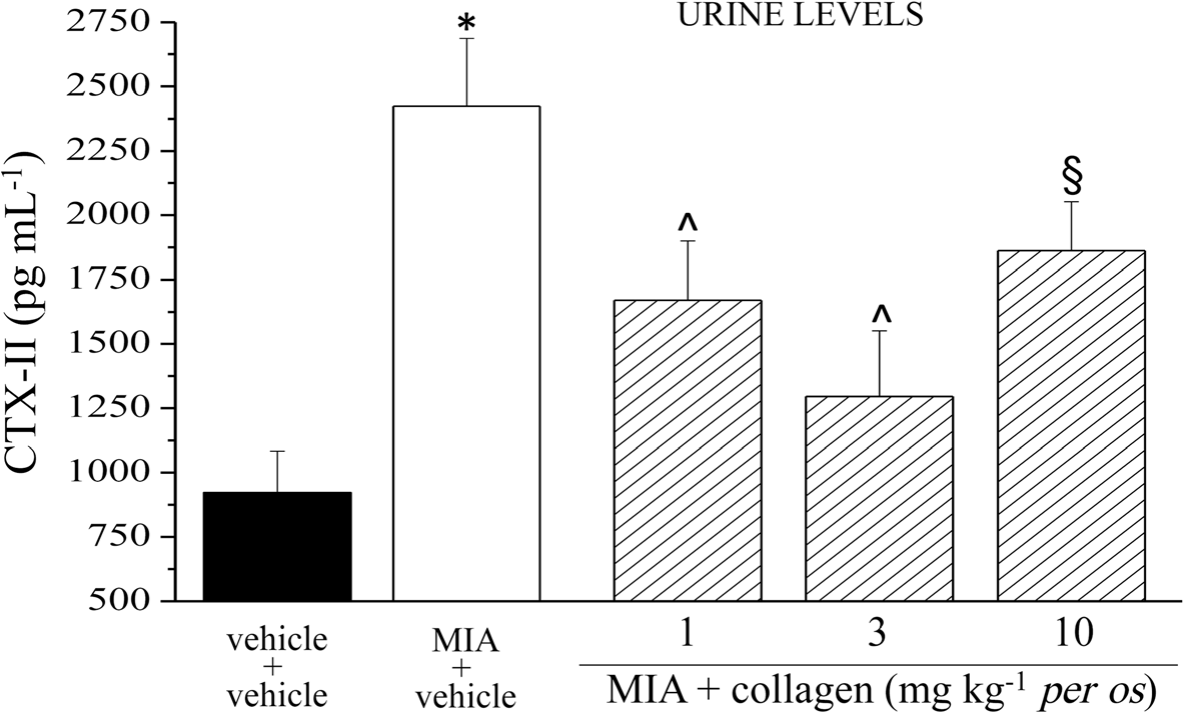
**CTX-II, urine levels.** On day 1, 2 mg MIA in 25 μl saline were delivered in the left knee articular cavity. Collagen was suspended in 1% CMC and p.o. daily administered starting on day 1. Control animals were treated with vehicle. On day 14 urine samples were collected and analyzed by ELISA kit. Each value represents the mean of 12 rats performed in 2 different experimental set. *P < 0.01 with respect to vehicle + vehicle rats; ^§^P < 0.05 and ^P < 0.01 compared MIA + vehicle treated rats.

Biochemical analysis of biological fluids performed 14 days after MIA injection, allowed to observe a 4 fold increase of CTX-II in plasma of MIA + vehicle rats compared to the control group (vehicle + vehicle). The plasmatic CTX-II increase was reduced by 53% in the rats treated with 1 mg kg^-1^ collagen and by 40% in those treated with the higher doses (Figure 6). The same parameter was increased by MIA also in urine (2.6 fold with respect to the control); 3 mg kg^-1^ collagen induced the higher protective effect (75% inhibition compared to MIA + vehicle; Figure 7). 1, 3 and 10 mg kg^-1^ collagen daily administered p.o. for 14 days, in the absence of articular damage, did not alter CTX-II levels in plasma and urine (data not shown). In Table 1 are shown the plasmatic levels of the collagen synthesis marker CPII. MIA-induced articular damage evoked an increase of CPII that is unaltered by collagen repeated treatments.

**Table 1:** CPII levels

**CPII LEVELS (ng mL**^**-1**^**)**					
		MIA			
	vehicle + vehicle	vehicle	collagen 1 mg kg^-1^	collagen3 mg kg^-1^	collagen10 mg kg^-1^
plasma	232 ± 12	454 ± 31*	500 ± 28	486 ± 23	505 ± 32

2 mg MIA/25 μl were delivered in the left knee articular cavity on day 1 Collagen was p.o. daily administered starting on the same day. On day 14 plasma samples were collected and analyzed by ELISA kit. Each value represents the mean of 6 rats performed in 2 different experimental set. *P<0.01 compared to vehicle + vehicle rats.

## Discussion

Osteoarthritis, also called degenerative joint disease, is a chronic pathology frequently seen in knee, hip, spine and hand causing pain, stiffness, decreased range of motion, and reduced quality of life for million people throughout the world [5]. It is by far the most widespread joint-affecting disease. According to the World Health Organization, osteoarthritis is the sixth-leading cause of disability in the world [30], being comparable to that of asthma [31,32]. It is estimated that it affects over 12% of the total population in the USA [33], compared with 0.6% for rheumatoid arthritis [34]. The prevalence of osteoarthritis increases with age because the condition is not spontaneously reversible [35]. Almost 9.6% of men and 18.0% of women ages 60 years in the world are thought to have symptomatic osteoarthritis [36]. Given the increasing incidence of osteoarthritis with age, the extended life expectancy observed in the Western world (for example 20% of the Italian population is age > 65 years; [37]) is expected to result in a progressively higher number of people affected by this pathology.

The usual management of patients with hip or knee osteoarthritis requires a combination of non-pharmacological and pharmacological modalities of therapy. Pharmacological treatments include acetaminophen, cyclooxygenase-2 (COX-2) non-selective and selective oral non-steroidal anti-inflammatory drugs (NSAIDs), topical NSAIDs and capsaicin, intra-articular injections of corticosteroids and hyaluronates, glucosamine and/or chondroitin sulphate for symptom relief. Glucosamine sulphate, chondroitin sulphate and diacerein have possible structure-modifying effects [38]. The characteristic of chronicity determines the need of new active disease modifying drugs.

In the present research osteoarthritis was mimicked in rats injecting MIA in the knee joint. The intra-articular injection of MIA induces necrosis of condrocytes with decrease of cartilage thickness and osteolysis [23], in the presence of a relevant component of oxidative stress [39]. Kobayashi et al. [40] showed that MIA is able to disorganize condrocytes and to promote cartilage erosion. These alterations are comparable with joint damages typical of humans affected by osteoarthritis [22,41,42].

In our experiments behavioral and biochemical features were evaluated 14 days after MIA injection, when pain as well as the degenerative articular process are overt [42,43]. At this time, CTX-II levels were strongly increased as measured in urine and plasma. CTX-II is a C-terminal peptide generated by the concerted action of matrix metalloproteinase (MMPs) on the fibrillar type II collagen, and it is considered a biomarker of cartilage degradation [44,45]. Its level was found to correlate with cartilage loss in animal models of osteoarthritis [46]. In agreement, clinical studies showed increased CTX-II levels in patients with osteoathritis compared with controls [47,48].

Type II collagen is the principal molecular component of mammalian cartilages [4]: the present work is focused on the study of this fibrous protein as preventive of MIA-induced articular damage. Different dosages of native type II collagen were daily administered per os for 14 days starting from the day of MIA injection. 1-10 mg dose range was chosen on the bases of the efficacy demonstrated in rheumatoid arthritis models [17]. Lower doses, in particular 1 mg kg^-1^, was able to strongly prevent pain behavior when evaluated both as an increase on suprathreshold stimulation (hyperalgesia-related measure) or as pain threshold decrease (allodynia-related measure). Collagen was able to reduce pain on the paw ipsilateral to the MIA injection. Since articular pain and joint tenderness are the most frequent and disabling symptoms [5] we evaluated also the animal responses to a direct stimulation of knee joint. The efficacy of collagen on reducing articular pain progressively increased lowering in the dose. Moreover, collagen-dependent pain relief allowed also to reduce postural unbalance, a feature of disease progression [26], as measured by hind limb weight bearing alterations. A general improvement of motor activity was observed.

The behavioral positive effects of collagen may be related to a prevention of articular damage since CTX-II levels were reduced in urine and in plasma of collagen-treated rats. On the other hand, low dosed collagen did not promote the synthesis of new collagen given that the plasmatic levels of CPII, the carboxyl propeptide of type II procollagen [28,29], was not altered by 14 days of collagen treatment.

The higher efficacy of the lower dose and, in general, the low dosages administered in the present work do not justify a mechanism founded on cartilage structure supplementation, as confirmed by the lack of CPII increase. Moreover, cartilage has limited repair capabilities and cartilage damage is difficult to heal since chondrocytes are bound in lacunae and they cannot migrate to damaged areas; hyaline cartilage does not have a blood supply and the deposition of new matrix is slow [49]. Other working mechanisms remain to be explored.

In rheumatoid arthritis the induction of oral tolerance was suggested as mechanism for the beneficial effect on pain evoked by low doses of native collagen [17,50]. The relevance of oral tolerance has been described for pathologies related to immune dysregulations and for autoimmune diseases [51], indeed a complex immune mechanism contribute to the pathology of rheumatoid arthritis [16,52]. The proposed mechanism for collagen-induced oral tolerance is that dendritic cells in the GALT take up the collagen and present it to T cells to generate regulatory T cells. Regulatory T cells control the immune response inducing several inhibitory cytokines, such as transforming growth factor β and interleukin 10, while decreasing pro-inflammatory cytokines [17,53].

To the actual knowledge osteoarthritis has not immune characteristic, but shares with rheumatoid arthritis cartilage degradation and the consequent inflammatory response. The role of oral tolerance in the management of osteoarthritis remains to be investigated. However, future research focused on the pharmacodynamic study of collagen therapeutic effects could offer new insights in osteoarthritis pathophysiology.

## Conclusions

Low dose collagen decrease pain induced by the intrarticular injection of MIA, a rat model of articular damage that mimics osteoarthritis alteration. Treated animals showed a reduced postural unbalance and a general improvement of motor activity. The decrease of CTX-II levels in urine and plasma suggests a protective effect on cartilage. This evidence highlights the interest for further investigation about the mechanism of low dose collagen and its relevance in osteoarthritis therapy.

## Competing interests

CG received a grant from MDM S.p.A, Monza Italy.

## Authors’ contributions

LDCM participated in the design of the study and drafted the manuscript. LM and MZ carried out the behavioral tests, the immunoassays and performed the statistical analysis. CG conceived the study, participated in its design and to draft the manuscript. All authors read and approved the final manuscript.

## Pre-publication history

The pre-publication history for this paper can be accessed here:

http://www.biomedcentral.com/1471-2474/14/228/prepub
